# A propos d'un cas rare de dacryocystite aiguë bilatérale chez un nourrisson

**DOI:** 10.11604/pamj.2015.20.71.6104

**Published:** 2015-01-27

**Authors:** Anoune Mariam, Tahri Hicham

**Affiliations:** 1 Service d'Ophtalmologie, CHU Hassan II, Fès, Maroc

**Keywords:** Dacryocystite aiguë, nourrisson, sac lacrymal, acute dacryocystitis, infant, lacrimal sac

## Image en medicine

La dacryocystite aiguë du nourrisson est un abcès aigu du sac lacrymal. Elle survient sur un sac lacrymal distendu. Cette complication très rare de l'imperméabilité lacrymo-nasale, survient lors des premiers mois de vie. Il s'agit d'un nourrisson de sexe féminin âgé de 50 jours, ayant comme antécédents un larmoiement clair puis devenu purulent depuis la naissance, qui se présente aux urgences ophtalmologiques avec une tuméfaction inflammatoire bilatérale en regard du sac lacrymal (A). Sur le plan général, le nourrisson est fébrile avec des signes de déshydratation. La tomodensitométrie montre deux images au niveau des 2 orbites hypodenses supéro-internes avec prise de contraste en périphérie (B). Cet aspect clinique et radiologique peut être en faveur de: dacryocystite aigue bilatérale sur sténose congénitale du canal lacrymo-nasal; dacryocèle bilatéral surinfecté; cellulite orbitaire compliquant une ethmoïdite. Le diagnostic de dacryocystite aigue bilatérale est retenu devant la présence d'un reflux purulent par les points lacrymaux à la pression digitale des deux collections. Le traitement consiste alors en une réhydratation par voie veineuse ainsi qu'une antibiothérapie par voie générale et locale, associées à une ponction des deux collections sous anesthésie générale et un sondage des voies lacrymales. L’évolution est favorable 48 heures après traitement (C).

**Figure 1:**
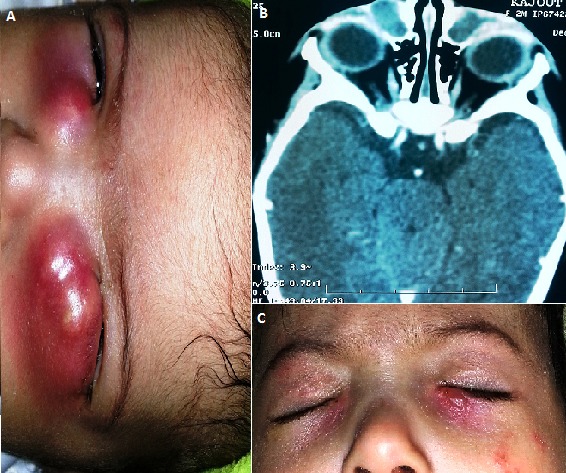
A) présence de deux collections abcédées en regard du sac lacrymal; B) coupe axiale d'une tomodensitométrie orbito-cérébrale objectivant deux images au niveau des 2 orbites hypodenses supéro-internes avec prise de contraste en périphérie; C) évolution après traitement

